# Integrating preoperative gait kinematics into total knee arthroplasty planning: Associations with intraoperative robotic laxities and prediction of postoperative function

**DOI:** 10.1002/jeo2.70826

**Published:** 2026-06-26

**Authors:** Jean Baltzer, Alix Cagnin, Julien Erard, Clément Favroul, Laurence Chèze, Nicola Hagemeister, Alex Fuentes, Elvire Servien, Cécile Batailler, Sébastien Lustig

**Affiliations:** ^1^ Orthopaedics Surgery and Sports Medicine Department, FIFA Medical Center of Excellence Croix‐Rousse Hospital, Hospices Civils de Lyon, Lyon North University Hospital Lyon France; ^2^ Emovi Montreal Quebec Canada; ^3^ Univ Lyon, Univ Gustave Eiffel, Univ Claude Bernard Lyon 1 Lyon France; ^4^ Research Centre of the Centre Hospitalier de l'Université de Montréal (CRCHUM) Montreal Quebec Canada; ^5^ Laboratoire de Recherche en Imagerie et Orthopédie de l'École de Technologie Supérieure (ÉTS) Montreal Quebec Canada

**Keywords:** dynamic alignment, functional knee positioning, gait analysis, intraoperative laxities, robotic‐assisted TKA, surgical planning

## Abstract

**Purpose:**

Total knee arthroplasty (TKA) is evolving from static measures towards function‐focused, robotic‐assisted personalisation. Integrating knee kinematics captured under weight‐bearing conditions may add clinically relevant information, but remains underexplored. This study aimed to explore the relevance of integrating preoperative knee dynamic coronal alignment during gait (mean varus‐valgus [V‐V] alignment during stance/swing) to better inform TKA planning.

**Methods:**

This prospective observational study enrolled 30 consecutive patients scheduled for robotic‐assisted TKA; 26 completed a preoperative 3D gait assessment (knee Kinesiography exam). Of these, 17 completed a 6‐month postoperative gait assessment. The primary analysis evaluated associations between preoperative dynamic V‐V alignment and intraoperative laxities (in extension and flexion) using Pearson correlations. The secondary analysis explored whether preoperative dynamic V‐V and intraoperative planned laxities jointly predicted postoperative dynamic V‐V alignment using multiple linear regressions in a varus morphotype subgroup (*n* = 13). Pearson correlations, Chi‐square, *T*‐tests were also used to evaluate associations between postoperative dynamic V‐V alignment and PROMs (satisfaction and knee osteoarthritis outcome score joint‐replacement [KOOS‐JR]).

**Results:**

The primary analysis (*n* = 26) reported that patients exhibiting more varus alignment during gait before TKA had significantly greater initial lateral laxity in extension, and smaller medial laxity in flexion (0.592 ≥ │*r*│≥0.475, *p* ≤ 0.03). Secondary analyses showed significant prediction of postoperative dynamic V‐V alignment with preoperative dynamic V‐V and robotic planned laxities in flexion as predictors (*R* ^2^ > 0.90, all *p* ≤ 0.04). Postoperative dynamic V‐V during stance was associated with KOOS‐JR scores (postoperative dynamic varus >2°, 94.4 vs. 73.2, *p* < 0.001).

**Conclusion:**

Results suggest that preoperative knee coronal kinematics during gait is linked to intraoperative laxities in TKA. Exploratory results further indicate associations between dynamic behaviour and outcomes, while suggesting that complementing robotic planned laxities with preoperative dynamic coronal alignment may enhance the ability to predict post‐TKA function in varus knees. Although additional studies will be needed, gait kinematics promise to help personalise TKA.

**Level of Evidence:**

Level IV, observational study.

**Abbreviations**
CS: cruciate‐substituting
HKA: hip‐knee‐ankle angle
KOOS‐JR: knee injury and osteoarthritis outcome score for joint replacement
PROMs: patient‐reported outcome measures
RMSE: root mean‐squared error
TKA: total knee arthroplasty
V‐V: varus‐valgus
WB: weight‐bearing

## INTRODUCTION

Coronal limb alignment is a key parameter when aiming for functional restoration and implant longevity in total knee arthroplasty (TKA) [19, 21]. It guides surgical planning and implant positioning, and influences load distribution across the tibial plateau, which can affect wear patterns and fixation stability [33]. However, recent studies highlighted the limitations of this parameter to impact patient‐reported TKA outcomes, joint forces during functional activities, and to globally capture dynamic joint behaviour [4, 29, 34]. Although surgical strategies usually aim for either neutral mechanical alignment or a patient‐specific anatomical approach, the alignment achieved during surgery often changes when weight is applied to the joint and during movement [5, 25]. Furthermore, standing coronal alignment after TKA shows limited predictive value for dynamic limb behaviour during gait [29]. This calls for an evolution from static measures to more function‐focused data, supporting the introduction of the notion of dynamic coronal alignment in TKA [28].

Dynamic varus‐valgus (V‐V) alignment captured during gait is influenced by multiple factors, including bony morphology, implant geometry, soft tissue behaviour and muscle forces [10, 24]. In addition, associations between dynamic V‐V alignment and patient‐reported outcome measures (PROMs) have been established in osteoarthritis patients, supporting further investigation of its relevance in the TKA context [2, 34]. Since dynamic V‐V alignment constitutes an actionable data, it could be used as a functional target in surgical planning. However, despite advances in imaging and navigation, the relationship between surgical choices and dynamic postoperative alignment remains incompletely understood.

Most studies exploring this question relied on static assessments or motion capture methods which are limited by soft tissue artefacts and lack precision in measuring V‐V angles [11]. A recent study investigated how surgical parameters in robotic‐assisted TKA influence postoperative dynamic V‐V knee alignment during daily activities, reporting significant but limited associations, explaining a small portion of the postoperative alignment variability [8]. However, this fluoroscopic study did not incorporate preoperative kinematic data, despite their potential influence on TKA outcomes [10, 34]. This leaves a gap in understanding the potential role of preoperative dynamic behaviour in TKA planning and in influencing postoperative dynamic V‐V alignment.

This study, therefore, aimed to explore the relevance of integrating preoperative knee dynamic V‐V alignment during gait (stance and swing phases) to better inform TKA planning. A primary analysis evaluated associations between preoperative dynamic V‐V alignment and robotic intraoperative laxity measures (both in extension and flexion). A secondary analysis explored whether preoperative dynamic V‐V and intraoperative planned laxities could jointly predict postoperative dynamic V‐V alignment after TKA.

## MATERIALS AND METHODS

### Patients

This was an observational, prospective study conducted on 30 consecutive patients receiving primary robotic‐assisted TKA between 1 June 2024, and 30 November 2024. This study was conducted using anonymised data obtained from routine clinical practice, in compliance with the French MR‐004 framework, with no intervention beyond standard care. During this period, three‐dimensional (3D) gait assessment was a systematic part of the standardised care pathway. Patients were eligible for inclusion if they presented with medial or global knee osteoarthritis (Ahlbäck score ≥ 2), demonstrated no coronal laxity (i.e., clinical integrity of the medial and lateral collateral ligaments), and provided written informed consent. Exclusion criteria were revision TKA, prior osteotomy on the affected knee, or inability to walk on a treadmill.

**Figure 1:**
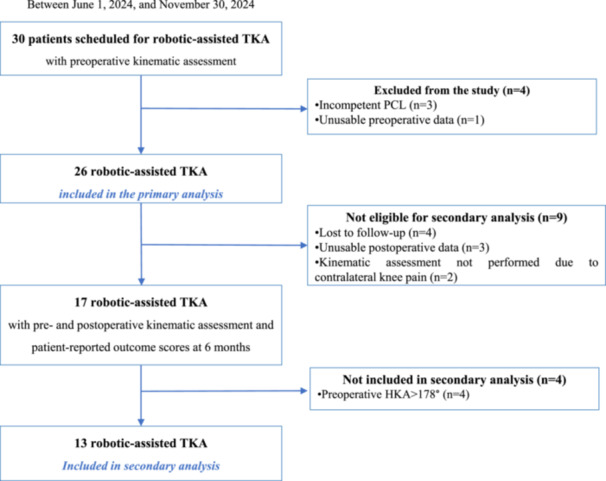
Flowchart. HKA, hip‐knee‐ankle angle; TKA, total knee arthroplasty.

The primary analysis included 26 patients who underwent surgery and completed a knee kinematic assessment during gait prior to surgery. Only 17 patients completed a follow‐up gait assessment 6 months post‐TKA, mainly due to follow‐up losses (see flowchart, Figure 1). Secondary analyses focused on a subgroup with a homogeneous preoperative morphotype (‘varus morphotype’: radiographic hip‐knee‐ankle [HKA] angle <178°; *n* = 13).

### Surgical technique

Surgery was performed by one experienced senior orthopaedic surgeon. The surgeon had prior experience using the implant (TRIATHLON cruciate‐substituting‐CS Total Knee Implant System). For all patients, posterior cruciate ligament was preserved, and the implant was positioned with an image‐based robotic assistance (MAKO, Stryker) using the functional knee positioning technique [15, 32]. All implants were uncemented.

**Figure 2:**
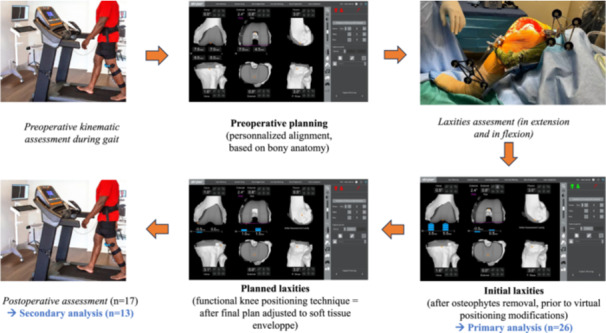
Overview of study phases.

Preoperative planning began with a personalised 3D plan based on the patient's individual anatomy. Knee laxities in extension (~10°) and flexion (~90°) were assessed intraoperatively using robotic assistance at two stages: after osteophytes removal but prior to implant virtual positioning modifications (‘initial laxities’) and after final implant virtual positioning (‘planned laxities’; Figure 2). Initial laxities guided 3D adjustments of implant positioning to achieve the desired final laxity profile. The surgeon aimed to balance the joint in extension (≤1 mm of medial and lateral gap), and a slightly greater lateral gap in flexion was intentionally targeted to account for the preservation of the PCL, ensuring physiological femoral roll‐back and avoiding over‐tensioning of the flexion gap. Recorded surgical parameters included medial and lateral gap measurements in both extension and flexion for the initial and final surgical plans, as well as planned femoral‐tibial implant component alignment.

Following surgery, all patients completed an identical, structured rehabilitation program. This comprehensive program consisted of three weekly sessions with a qualified physiotherapist, over a period of 2–3 months. During these sessions, patients performed targeted stretching exercises to improve knee flexion‐extension motion and prevent muscle stiffness.

### Kinematic assessment

**Figure 3:**
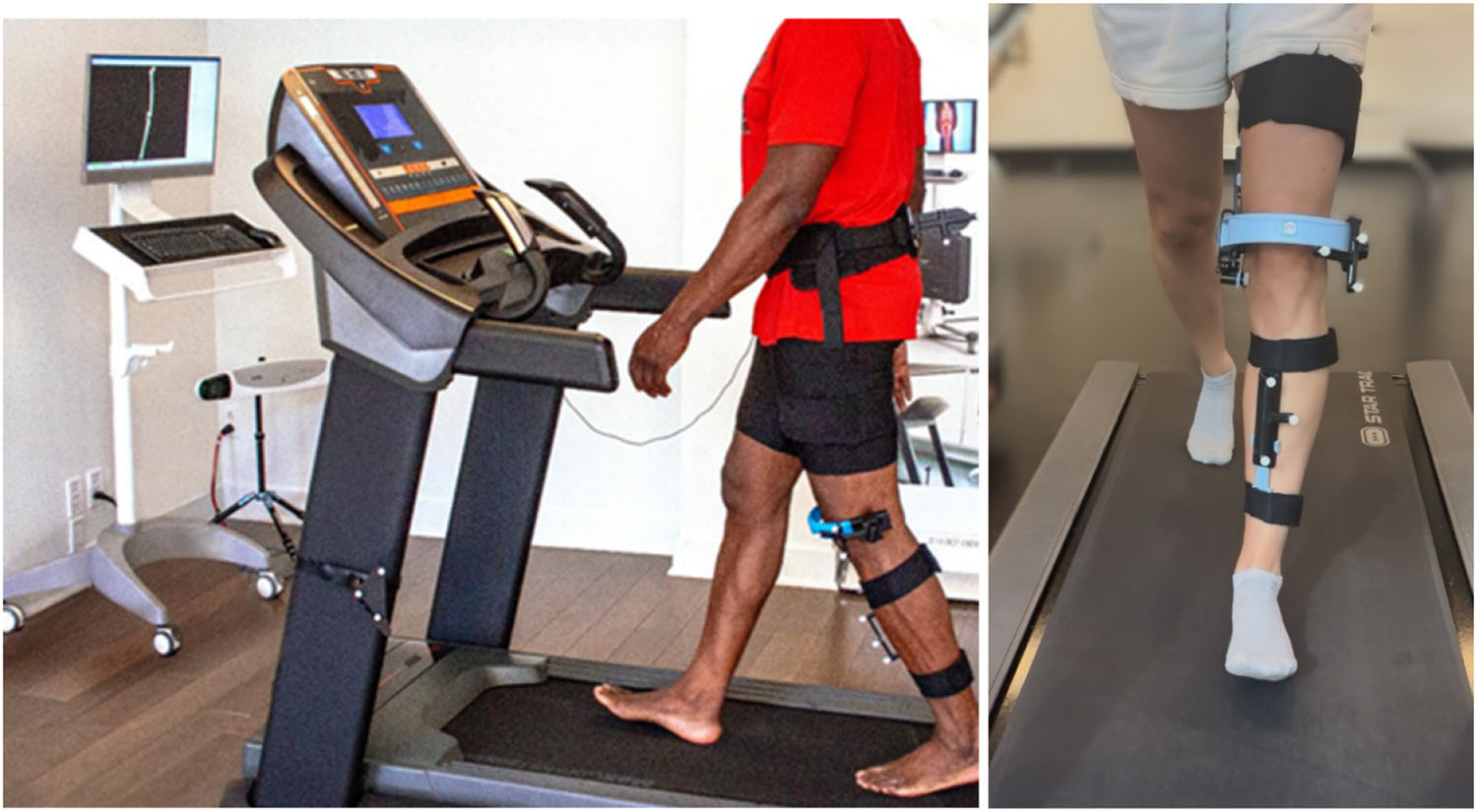
Knee kinematics during gait were captured in clinic before and 6 months after TKA using the KneeKG® system. TKA, hip‐knee‐ankle angle.

All patients performed a knee kinesiography exam preoperatively, while 17 (65%) completed a follow‐up assessment at 6 months (±2 weeks) postoperatively, using the KneeKG® system (Emovi Inc.) during treadmill walking [7, 18]. This is a non‐invasive medical device designed for 3D analysis of dynamic knee motion under full weight‐bearing (WB) conditions. It consists of an exoskeleton (including a femoral arch, tibial sensor attachments and a sacral belt), an infrared camera (a Polaris Spectra camera from Northern Digital Inc.) and a computer with Knee3D™ software (Emovi Inc.) (Figure 3). The system enables the measurement of knee dynamic V‐V alignment with an accuracy of 0.4°, compared to the measurement of bony motion by fluoroscopy, with high intra‐/inter‐observer reliability (intraclass correlation coefficients [ICCs] ≥ 0.92) [18].

The recording protocol was done according to the technique described by Bytyqi et al. [3].

The same protocol was applied in both preoperative and postoperative assessments. The dynamic V‐V alignment during stance (i.e., mean value between 20% and 54% of the gait cycle, from flat foot on the ground to terminal stance; WB) and during swing (i.e., maximum value between 70% and 90%; non‐WB) were calculated [26].

### Clinical and radiographic data

Patients underwent standardised postoperative clinical follow‐ups at 2 and 6 months after surgery by the same surgeon. The knee injury and osteoarthritis outcome score for joint replacement (KOOS‐JR) and patient satisfaction were recorded at 6 months to document PROMs [30]. Radiographic evaluations performed before and 6 months postoperatively included anteroposterior, lateral, patellar axial and standing long‐leg films (bipedal stance). All radiographs were reviewed by the same surgeon who performed the surgery (non‐blinded) using Centricity Universal Viewer Zero Footprint (version 6.0 SP7.0.2, GE Healthcare), a system capable of measuring angles with an accuracy of 0.1°. These evaluations included pre‐ and postoperative measurements of the HKA angle. Figure 2 summarises the sequential stages of the study design and implementation.

### Statistical analysis

Descriptive patient characteristics were reported using mean values, standard deviations (SD) and range (minimum; maximum).

Bivariate Pearson correlations were used to evaluate the associations between preoperative V‐V dynamic alignment and robotic intraoperative initial laxities (primary analysis). Associations were evaluated between preoperative V‐V alignment during stance and during swing with initial intraoperative laxities including medial‐lateral asymmetries (i.e., difference between medial and lateral laxities) in extension and in flexion, as well as extension‐to‐flexion imbalances (i.e., difference between extension and flexion laxities) medially and laterally.

Multiple linear regressions were used to explore whether preoperative V‐V dynamic alignment and intraoperative planned laxities could jointly predict knee dynamic behaviour post‐TKA (secondary analysis). Two regression models were tested to predict postoperative V‐V alignment during stance. The first model (M1‐stance) included four planned intraoperative parameters as predictor variables (medial gap in extension, medial gap in flexion, lateral gap in extension, lateral gap in flexion) and the implant component alignment (femoral + tibial) as a control variable. Laxity asymmetries and imbalances were not included to avoid multicollinearity issues. The second model (M2‐stance) retained the same predictors and covariate as M1‐stance, and integrated preoperative V‐V alignment during stance as an additional predictor variable.

Two similar models (M1‐swing and M2‐swing) were used to predict postoperative V‐V alignment during swing (i.e., with four intraoperative predictor variables, and with the addition of preoperative V‐V alignment during swing). Predictor variables were all introduced in the models at first, and progressively removed following a backward elimination method (i.e., iterative removal of the least influential predictor with a criterion at F‐to‐remove probability ≥0.1). Models’ performance was assessed using the adjusted *R*‐squared (*R* ^2^) to account for the number of predictors and root mean‐squared error (RMSE) in order to identify the best multi‐predictor model possible to predict postoperative V‐V alignment during stance and swing. To complement this secondary analysis, bivariate Pearson correlations, Chi‐square and independent T‐tests were used to explore associations between postoperative V‐V alignment during gait and PROMs (i.e., KOOS‐JR and satisfaction).

Considering that these regression models aim to explore associations between the correction of kinematic deficiencies post‐TKA and intraoperative predictors, there was a necessity to apply these models independently on similar morphotypes (i.e., static varus/valgus type of knees). This is supported by multiple studies identifying significant kinematic differences between varus and valgus deformities in healthy knees, before, and after TKA [1, 2, 3, 4, 5, 6, 7, 8, 9, 10, 11, 12, 13, 14, 15, 16], reporting different needs in terms of soft‐tissue releases [28], and further differences in function restoration and outcomes [19].

A *p*‐value < 0.05 was considered statistically significant for all analyses. All statistical analyses were conducted using IBM SPSS Statistics for Windows version 31.0 (IBM Corp.) [8].

## RESULTS

### Primary analysis

Associations between preoperative V‐V alignment during gait and robotic initial laxities (*n* = 26).

Out of the 30 participants, 26 completed the preoperative assessment and were included in the primary analysis (Figure 1). Their mean age at surgery was 69.7 ± 9.0 years (54.0; 86.0), and 61.5% were men. The mean preoperative HKA angle was 173.8° ± 4.5 (167; 182).

**Figure 4:**
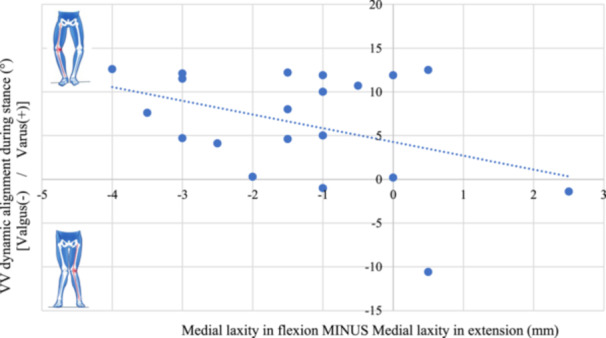
Significant association between preimplantation intraoperative laxities (extension‐to‐flexion imbalance on the medial side) and V‐V dynamic alignment during stance. VV, varus‐valgus.

Mean values of initial intraoperative laxities and their associations with preoperative V‐V dynamic alignment during stance and during swing are presented in Table 1. Knee V‐V dynamic alignment before surgery was moderately to strongly associated with initial intraoperative laxities throughout gait (0.458 ≤ |*r* | ≤0.592, all *p* ≤ 0.04). Patients exhibiting more varus dynamic alignment pre‐surgery had significantly greater lateral laxity in extension, and smaller medial laxity in flexion (especially compared to their lateral side, asymmetry in flexion: *r* > 0.475, *p* = 0.03). Interestingly, extension‐to‐flexion laxity imbalance on the medial side emerged as the intraoperative measure most strongly associated with preoperative dynamic V‐V alignment (during stance and swing, both *p* ≤ 0.01, both │*r*│≥0.548; Table 1 and Figure 4). From a surgical planning perspective, knees presenting with increased dynamic varus exhibited significantly greater medial compartment tightness during intraoperative flexion (i.e., flexion laxity < extension laxity, *r* = −0.592; Table 1).

**Table 1:** Mean values of initial intraoperative laxities and their associations with preoperative V‐V dynamic alignment during stance and during swing.

	Mean values (95% confidence interval)	V‐V alignment during stance (WB)	V‐V alignment during swing (non‐WB)
	mm	Pearson *r* (*p*‐value)	Pearson *r* (*p*‐value)
Medial side			
Extension laxity	2.4 (1.7;3.0)	0.306 (*p* = 0.19)	0.154 (*p* = 0.52)
Flexion laxity*	1.1 (0.4;1.8)	−0.317 (*p* = 0.17)	−0.406 (*p* = 0.08)¨
Extension‐to‐flexion laxity imbalance***	−1.3 (−2.0;−0.5)	−0.592 (*p* = 0.006)***	−0.548 (*p* = 0.01)***
**Lateral side**			
Extension laxity**	3.3 (2.6;4.0)	0.393 (0.09)*	0.458 (0.04)**
Flexion laxity	2.4 (1.6;3.2)	0.195 (*p* = 0.41)	0.09 (*p* = 0.70)
Extension‐to‐flexion laxity imbalance	−0.9 (−1.8;0.0)	−0.136 (0.57)	−0.279 (0.23)
**Asymmetry in extension**			
Lateral/medial difference	0.9 (0.3;1.5)	0.130 (0.59)	0.364 (*p* = 0.11)
**Asymmetry in flexion**			
Lateral/medial difference**	1.3 (0.5;2.1)	0.496 (*p* = 0.03)**	0.475 (*p* = 0.03)**

*Note*: Extension‐to‐flexion laxity imbalance: Flexion laxity MINUS extension laxity, that is, positive value means flexion laxity > extension laxity; lateral/medial asymmetry: lateral laxity MINUS Medial laxity, that is, positive value means Lateral laxity > medial laxity. *p ≤ 0.1; **p ≤ 0.05; ***p ≤ 0.01.

Abbreviations: V‐V, varus‐valgus; WB, weight‐bearing.

### Secondary analysis

Combination of preoperative V‐V alignment during gait and robotic planned parameters to predict postoperative dynamic alignment (*n* = 13).

Focusing on preoperative varus morphotype knees (*n* = 13), results of the multiple linear regression models’ to predict postoperative V‐V alignment during stance and during swing are presented in Tables 2 and 3.

**Table 2:** Results of multiple linear regressions predicting postoperative varus‐valgus (V‐V) alignment during stance.

	*B*	SE	*β*	*p*	Retained/removed
**M1‐stance final model**					
Covariate					
Component_VV (° of varus)	1.19	0.63	0.48	0.09	
Predictors					
Ext_LATERAL (mm)	—	—	—	—	*Removed at step 1 (p* = *0.90)*
Ext_MEDIAL (mm)	—	—	—	—	*Removed at step 2 (p* = *0.79)*
Flex_LATERAL (mm)	−8.21	4.75	−1.61	0.12	
Flex_MEDIAL (mm)	9.27	4.88	1.8	0.09	
Initial model (all predictors included): *R* ^2^ = 0.065 | RMSE = 4.7 | *p* = 0.41; Step1: *R* ^2^ = 0.180 | RMSE = 4.4 | *p* = 0.25;					
Step 2 (final model): *R* ^2^ = 0.264 | RMSE = 4.2 | *p* = 0.13.					

**M2‐stance final model**					
Covariate					
Component_VV (° of varus)	0.59	0.31	0.23	0.10	
Predictors					
Ext_LATERAL (mm)	—	—	—	—	*Removed at step 2 (p* = *0.64)*
Ext_MEDIAL (mm)	—	—	—	—	*Removed at step 1 (p* = *0.34)*
Flex_LATERAL (mm)	−8.31	2.22	−1.63	0.006*	
Flex_MEDIAL (mm)	12.4	2.35	2.37	<0.001*	
Preop_VV_stance (° of varus)	1.42	0.25	0.90	<0.001*	
Initial model (all predictors included): *R* ^2^ = 0.825 | RMSE = 2.0 | *p* = 0.006*; Step1: *R* ^2^ = 0.822 | RMSE = 2.40 | *p* = 0.002*.					
Step 2 (final model): *R* ^2^ = 0.839 | RMSE = 1.9 | *p* < 0.001*					

*Note*: *B* = unstandardised coefficient; SE = standard error; *β* = standardised coefficient; *R* ^2^ = adjusted *R*‐square; Component_VV = femoral + tibial implant component varus‐valgus alignment; Ext_LATERAL = lateral laxity in extension; Ext_MEDIAL = medial laxity in extension; Flex_LATERAL = lateral laxity in flexion; Flex_MEDIAL = medial laxity in flexion; Preop_VV_stance = preoperative dynamic varus‐valgus during stance; Covariate was retained in the final model regardless of significance.

**Table 3:** Results of multiple linear regressions predicting postoperative varus‐valgus (V‐V) alignment during swing.

	*B*	SE	*β*	*p*	Retained/removed
**M1‐swing final model**					
Covariate					
Component_VV (° of varus)	0.32	0.79	0.12	0.70	
Predictors					
Ext_LATERAL (mm)	—	—	—	—	*Removed at step 1 (p* = *0.63)*
Ext_MEDIAL (mm)	−8.10	4.73	−1.79	0.12	
Flex_LATERAL (mm)	—	—	—	—	*Removed at step 2 (p* = *0.34)*
Flex_MEDIAL (mm)	9.68	6.02	1.70	0.14	
Initial model (all predictors included): *R* ^2^ = −0.058 | RMSE = 5.4 | *p* = 0.55; Step 1: *R* ^2^ = 0.041 | RMSE = 5.2 | *p* = 0.41;					
Step 2 (final model): *R* ^2^ = 0.040 | RMSE = 5.2 | *p* = 0.38					

**M2‐swing final model**					
Covariate					
Component_VV (° of varus)	−0.27	0.55	−0.10	0.64	
Predictors					
Ext_LATERAL (mm)	6.19	2.95	1.53	0.08	
Ext_MEDIAL (mm)	−12.24	4.50	−2.72	0.04*	
Flex_LATERAL (mm)	−12.02	4.32	−2.17	0.03*	
Flex_MEDIAL (mm)	20.56	5.39	3.60	0.01*	
Preop_VV_swing (° of varus)	0.81	0.23	0.80	0.01*	
Initial model (all predictors included – final model): *R* ^2^ = 0.606 | RMSE = 3.3 | *p* = 0.05*					

*Note*: *B* = unstandardised coefficient; SE = standard error; *β* = standardised coefficient; *R* ^2^ = adjusted *R*‐square; Component_VV = femoral + tibial implant component varus‐valgus alignment; Ext_LATERAL = lateral laxity in extension; Ext_MEDIAL = medial laxity in extension; Flex_LATERAL = lateral laxity in flexion; Flex_MEDIAL = medial laxity in flexion; Preop_VV_swing = preoperative dynamic varus‐valgus during swing; Covariate was retained in the final model regardless of significance.

The M1‐stance final model failed to reach statistical significance and showed high RMSE (>4°) indicating insufficient performance. Planned laxities alone (in flexion) were not influential predictors when controlled for the implant component alignment (best model *R* ^2^ = 0.264, *p* = 0.13). Integrating preoperative dynamic V‐V alignment strongly enhanced prediction performance with statistically significant M2‐stance models explaining up to >80% of the variance of postoperative dynamic V‐V alignment during stance (all *p* < 0.01). The best model combined preoperative V‐V alignment during stance with both planned medial and lateral laxities in flexion, once controlled for the final implant component alignment (*R* ^2^ = 0.839, *p* < 0.001). This M2‐stance model also showed good RMSE (i.e., <2°). Using standardised Beta coefficients, it was found that postoperative alignment during stance increased by 0.9° of varus for each degree of preoperative varus alignment during stance, by 2.4° for each millimetre increase of medial laxity in flexion. Conversely, postoperative alignment decreased by −1.6° (i.e., towards valgus) for each millimetre increase in lateral laxity in flexion (*p* ≤ 0.006 for all three coefficients, Table 2).

Regression models predicting postoperative V‐V alignment during swing showed similar results, with M1‐swing models failing to reach statistical significance (*p* = 0.38). M2‐swing models including preoperative V‐V alignment during swing explained more than 80% of variance, with slightly higher RMSE (3.3°, *p* ≤ 0.05). However, all potential predictors were retained in the model, which led to a lower *R* ^2^ (0.606). The best model indicated that postoperative alignment during swing increased by 0.8° of varus for each degree of preoperative varus alignment during swing, by 3.6° and 1.5° for each millimetre increase of medial laxity in flexion and lateral laxity in extension respectively. Conversely, postoperative alignment decreased by −2.7° and −2.2° (i.e., towards valgus) for each millimetre increase of medial laxity in extension and lateral laxity in flexion respectively (*p* < 0.08 for all five standardised Beta coefficients, Table 3).

Specifically for preoperative varus morphotype knees (*n* = 13), postoperative V‐V alignment during stance was very strongly associated with KOOS‐JR scores (*r* = 0.737, *p* = 0.004). From a clinical point‐of‐view, more varus alignment dynamically was associated with better function. Notably, patients who exhibited a residual varus alignment during stance post‐TKA (>2°, *n* = 5) reported significantly higher KOOS‐JR scores 6 months after their surgery (94.4 vs. 73.2, *p* < 0.001). Although these patients also appeared to be more likely to be very satisfied with their surgery (*n* = 5/5: 100%), the difference was not significant (postoperative V‐V alignment during stance <2°: *n* = 5/8 = 63% were very satisfied, *p* = 0.12). Interestingly, the association between PROMs and V‐V alignment during swing was not significant (*r* = 0.319, *p* = 0.29). All complementary results are presented in detail in Table 4. Notably, while patients improved their walking speed by 0.24 ± 0.23 m/s (range: 0.00–0.61 m/s), postoperative walking speed was not associated with postoperative V‐V alignment nor KOOS‐JR scores (all *p* ≥ 0.27).

**Table 4:** Complementary results on preoperative varus morphotype knees.

Varus morphotype (*n* = 13)	Pre‐op	Post‐op	Delta
Patients’ characteristics			
Age (years)	71.2 ± 7.9	n.a	n.a
Sex (% males)	61.5%	n.a	n.a
HKA (°)	171.1 ± 2.6	177.4 ± 2.3	6.2 ± 2.7
Measures			
VV alignment during stance (°)	9.6 ± 3.1	2.4 ± 4.8	−7.3 ± 4.3
VV alignment during swing (°)	11.3 ± 5.2	7.5 ± 5.3	−3.8 ± 5.0
Gait speed (m/s)	0.78 ± 0.28	1.02 ± 0.16	0.24 ± 0.23
KOOS‐JR	n.a	81.4 ± 12.8	n.a
Satisfaction (% of patients)			
Very satisfied	n.a	77%	n.a
Satisfied	n.a	15%	n.a
Slightly satisfied	n.a	8%	n.a
Slightly unsatisfied	n.a	0%	n.a
Unsatisfied	n.a	0%	n.a
Very unsatisfied	n.a	0%	n.a

*Note*: Knees presenting with different preoperative morphotypes (three neutral and one valgus) included two (50%) patients reporting being very satisfied, two (50%) being satisfied, and an average KOOS‐JR score of 77.5 ± 18.4 (59.4;100).

Abbreviations: HKA, hip‐knee‐ankle angle; KOOS‐JR, knee injury and osteoarthritis outcome score for joint replacement; m/s, metres per second; n.a, non‐applicable.

## DISCUSSION

The study results support the relevance of integrating preoperative knee kinematic assessment during gait in informing TKA planning. Specifically, dynamic knee V‐V alignment preoperatively showed moderate to strong associations with initial intraoperative laxities across different gait phases (all *p* < 0.04). Individuals walking with preoperative dynamic varus presented greater lateral knee laxity in extension and reduced medial laxity in flexion.

Furthermore, exploratory analysis in the varus morphotype subgroup suggest that combining preoperative dynamic V‐V alignment to intraoperative robotic laxity measures may substantially improve the prediction of postoperative dynamic V‐V alignment after TKA. Additionally, patients in such morphotype appeared to achieve higher KOOS‐JR scores when they maintained a postoperative dynamic varus during stance (i.e., >2°; *p* < 0.001) [31]. These findings highlight the promising role of evaluating and regulating dynamic alignment during WB functional tasks in order to improve planning, define personalised targets and restore joint function.

Limited evidence is currently available regarding the relationship between preoperative knee kinematics and intraoperative laxity measurements obtained through robotic‐assisted procedures. This study reveals that dynamic coronal alignment during gait is significantly associated with intraoperative laxities, especially in flexion. While this result aligns with clinical biomechanical expectations, this is an important demonstration given that static measures of varus deformity appeared to be associated with extension laxities but have failed to anticipate flexion gap laxities in TKA [9]. These findings underscore the clinical value of integrating preoperative functional data into surgical planning, as it can help understanding the ligamentous situation, facilitating more accurate initial implant positioning, and may reduce reliance on intraoperative adjustments.

Several studies explored relationships between postoperative gait biomechanics and either PROMs or intraoperative laxities after TKA, providing relevant context for the present findings. In their comparative study involving patients with painful TKA, asymptomatic TKA, and healthy controls, Planckaert et al. identified three biomechanical features potentially contributing to unexplained postoperative pain: persistent flexion contracture during gait, V‐V alignment in stance phase, and a slight internal rotation of the combined prosthetic components—all of which are known to impact patellofemoral joint stress [27]. This aligns with the secondary analysis: in patients with a constitutional varus morphotype, those retaining dynamic varus during stance after TKA scored substantially higher on the KOOS‐JR (a 21‐point difference). Kurihara et al. also investigated the relationship between early postoperative gait biomechanics and patient‐reported outcomes 6 months after TKA [16]. They found that greater internal knee extension moment and negative joint power during early stance were moderately associated with better PROMs.

In another study, Kaneko et al. evaluated the impact of intraoperative and postoperative coronal knee laxity (specifically medial and lateral joint gaps at various flexion angles) on PROMs following anatomical bi‐cruciate retaining TKA for varus knees. They found that greater medial laxity at 90° of flexion was positively associated with improved patient satisfaction and expectations [13]. However, a distinction must be made between passive intraoperative laxity measurements and joint behaviour under physiological weight‐bearing conditions. Whereas Kaneko et al. reported that intraoperative medial laxity at 90° may facilitate high‐flexion activities such as deep flexion, the results of the present study suggest that maintaining dynamic varus alignment during gait (from 0° to 60° of flexion), and especially during stance, is important for postoperative functional satisfaction in patients with a constitutional varus morphotype. This suggests that a ‘tight’ but balanced medial compartment in a dynamic state may prevent instability and better mimic native biomechanics, whereas excessive laxity during physiological loading could lead to less predictable functional performance. In fact, a recent multi‐centre study suggested that excessive medial laxity, particularly in flexion, is associated with inferior clinical outcomes [22]. Still, in both studies gap assessment was performed using a tensor device and not with robotic assistance, which could be considered as a possible source of bias when drawing comparisons.

To ensure the relevance and the robustness of the secondary analyses, patients were grouped according to their preoperative coronal morphotype. This decision was driven by previous literature demonstrating substantial kinematic differences between these varus and valgus deformity types in native knees, as well as pre‐ and post‐TKA [1, 20]. Moreover, varus and valgus knees have been shown to require distinct soft‐tissue balancing strategies [35], and exhibit divergent patterns in functional recovery and clinical outcomes [23]. In addition, robotic planned laxities were used as predictors of postoperative dynamic alignment rather than post‐implantation laxities. Although such laxities could have better represented the final intervention of the surgery, they were not remeasured. This choice was driven by a recent study by Erard et al. demonstrating that planned laxities reliably reflect implanted soft‐tissue balance [6], and to avoid undue stress on uncemented implants.

These specific results build upon and extend the findings of Higinbotham et al. by demonstrating that integrating preoperative dynamic V–V alignment into intraoperative planning may substantially enhance the prediction of postoperative dynamic coronal knee behaviour. While Higinbotham et al. identified intraoperative medial flexion gap and implant alignment parameters as key predictors of dynamic V–V alignment 1 year postoperatively, their models exhibited limited statistical significance and moderate predictive performance (*R* ^2^ = 0.30 during gait) [10]. In contrast, the regression models of the present study incorporating preoperative dynamic V–V alignment achieved markedly superior performance, with the best‐fitting model explaining over 80% of the variance (M2‐stance: *R* ^2^ = 0.839, *p* < 0.001) and RMSE values close to 2°. While the limited sample size relative to the numbers of predictors must be taken in consideration (i.e., potential risk of overfitting), the most predictive model combined preoperative dynamic alignment during the weight‐bearing phase of gait (i.e., stance) with medial and lateral flexion laxities, once controlled for implant alignment. This resonates with findings from recent studies suggesting that preoperative dynamic V‐V behaviour influences postoperative dynamic alignment [17]. Such preoperative data should therefore be considered during surgical planning and intraoperative intervention to aim for a better control of the dynamic behaviour delivered post‐TKA.

Considered together, the findings support the hypothesis that integrating preoperative dynamic V‐V alignment could guide planning and intraoperative decision‐making in order to deliver knees with a residual dynamic varus alignment after TKA, and improve outcomes in patients with varus morphotypes. Nonetheless, they suggest that preoperative kinematic assessment captures patient‐specific mechanics, including soft tissue behaviour, that is not fully reflected in intraoperative static assessments. Yet, despite the integration of robotic assistance, intraoperative laxity measurements remain partially subjective due to non‐standardised manual force application, which may inadvertently affect coronal alignment outcomes postoperatively [12].

This study has several limitations that warrant consideration. The relatively small sample size may restrict the generalisability of the findings; however, this drawback is partly mitigated by the prospective design and the biomechanical nature of the investigation, which enabled precise and standardised data acquisition. Nonetheless, the generalisability of the findings is constrained by the single‐centre, single‐surgeon, single‐robot and single‐implant nature of the study. While this design ensured high technical standardisation for the kinematic analysis, it remains unclear whether the associations observed through regression models between planned laxities and dynamic gait patterns would persist across different prosthetic designs or alignment philosophies. Considering the limited sample size, there is an inherent risk of overfitting, and these associations will need to be re‐evaluated on unseen data to be confirmed. Another limitation of this study is the 6‐month follow‐up for gait analysis. While specific comparative data between 6‐month and long‐term kinematics are sparse, current clinical practice suggests that functional scores are achieved by this stage [14]. Additionally, secondary analyses (including prediction models) were restricted to knees with preoperative varus morphotype. Although this approach was primarily adopted for biomechanical consistency in assessing joint behaviour, results cannot be generalised for valgus knee morphotypes. Nevertheless, the strength and consistency of the results in varus knees highlight the need for prospective studies to determine whether preoperative dynamic V‐V alignment can also predict intraoperative and postoperative behaviour in valgus‐aligned knees, ideally across more diverse surgical techniques and robotic systems as well. Since function is 3D, other planes of movement of interest could also affect patients’ performance (e.g., sagittal plane movements of the CS component) and will need to be included in future prediction models/studies. Finally, the radiographic measurements were conducted by a single surgeon (main author) without blinding. Although a standardised digital measurement protocol was strictly followed to ensure accuracy, this represents a potential source of observer bias.

## CONCLUSIONS

Integrating preoperative dynamic alignment during gait in TKA planning could help anticipate intraoperative laxity and improve understanding of how surgical planning shapes postoperative dynamic coronal alignment. Results suggest that knees presenting with increased preoperative dynamic varus tend to exhibit significantly greater medial compartment tightness and lateral laxity during robotic‐assisted TKA. Furthermore, combining preoperative dynamic varus‐valgus and planned intraoperative laxities may substantially enhance dynamic coronal alignment prediction after robotic‐assisted TKA. In this exploratory cohort, preoperative varus morphotype knees who maintained a dynamic varus alignment during stance after TKA appeared to be associated with better PROMs, suggesting associations between postoperative gait kinematics and outcomes. External validation in larger, multicenter cohorts with more diverse alignment profiles is required to confirm the clinical relevance and generalisability of these findings to broader TKA populations. Considered together, study results support the relevance of incorporating preoperative weight‐bearing kinematics into TKA planning and intraoperative procedure to improve future personalised surgical procedures.

## AUTHOR CONTRIBUTIONS

**Jean Baltzer**: Study design; data collection; literature review and manuscript writing. **Alix Cagnin**: Study design; data collection; statistical analysis; literature review; manuscript editing. **Julien Erard**: Data collection and manuscript editing. **Clément Favroul**: Data collection and manuscript editing. **Laurence Chèze**: Study design and manuscript editing. **Nicola Hagemeister**: Study design and manuscript editing. **Alex Fuentes**: Study design and manuscript editing. **Elvire Servien**: Study design and manuscript editing. **Cécile Batailler**: Study design; supervision; literature review and manuscript editing. **Sébastien Lustig**: Study design; supervision; literature review and manuscript editing. All authors read and approved the final manuscript.

## FUNDING INFORMATION

The authors have no funding to report.

## CONFLICT OF INTEREST STATEMENT

Alix Cagnin: Paid employee/consultant for Emovi. Alex Fuentes: Paid employee/consultant for Emovi. Cécile Batailler: Consultant for Stryker, Lepine, Smith Nephew. Elvire Servien: Consultant for Corin. Sébastien Lustig: Consultant for Stryker, Smith Nephew, Heraeus, Depuy Synthes; Institutional research support from Groupe Lepine, Amplitude; Editorial Board for Journal of Bone and Joint Surgery (Am). The remaining authors declare no conflicts of interest.

## ETHICS STATEMENT

This study was conducted using anonymized data obtained from routine clinical practice, with no intervention beyond standard care. The written informed consent of the patients was collected by the investigating physician.

## Data Availability

The data that support the findings of this study are available from the corresponding author upon reasonable request.
